# Precision medicine and monoclonal antibodies: breach of promise?

**DOI:** 10.3325/cmj.2019.60.284

**Published:** 2019-06

**Authors:** Livio Garattini, Anna Padula

**Affiliations:** Institute for Pharmacological Research Mario Negri IRCCS, Ranica (BG), Italy *livio.garattini@marionegri.it*

Precision medicine (PM) is an emerging approach for disease treatment and prevention that, beyond environment and lifestyle, mainly takes into account individual gene variability (1). PM best incorporates all the expectations raised by the most advanced pharmacological therapies in development (2). Unlike competing definitions (eg, personalized medicine) (3), PM aims to stratify (rather than individualize) pharmacological therapies to subgroups of patients who have the genetic variant of interest (4), overcoming the traditional “one size fits all” drug paradigm. Pharmacogenomics − the study of the influence of genetic variability on drug responses (5) − should help identify “the right drug at the right dose for the right patient” (2). By influencing or predicting the response to treatment (1), PM should optimize the efficacy and safety of drugs administered according to the patient’s genomic profile (6), ideally maximizing pharmacological responses and minimizing the side effects (7). Since tumors tend to arise from genetic variants (1), cancer treatment has so far been the most investigated area of PM. However, longer-term expectations of PM are pharmaceutical therapies for all diseases (2).

Monoclonal antibodies (mABs) are the latest generation of drugs that fit the PM paradigm most (8). Approved in record numbers (at twice the rate of small molecules) (9) and having sky-high prices, mABs are indicated for many tumors (their main field of application) and chronic illnesses. The pharmaceutical industry expects biological markers – molecules found in the human body that are signs of normal or abnormal processes (10) − to play a major role in using mABs for optimal treatment in clinical practice (11). The first paradigmatic example of targeted therapy in oncology was trastuzumab (with HER2 as a biomarker in breast cancer) (12), followed by cetuximab (with EGFR and KRAS in colorectal cancer) (3).

To assess the general trend of mABs and their relationship with PM, we analyzed the main characteristics of the 68 mABs approved in the European Union (EU) in the last two decades (1998-2018).

## Monoclonal antibodies survey

**Figure 1:**
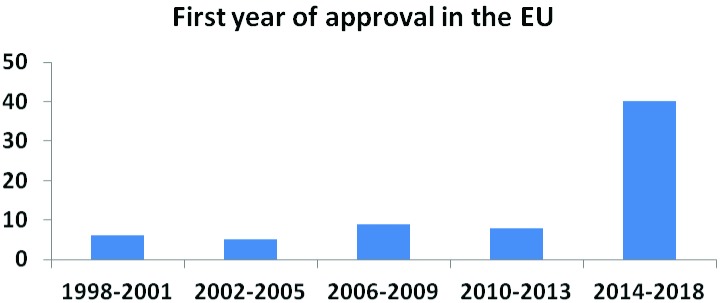
Monoclonal antibodies approved in the European Union (EU) by years.

**Figure 2:**
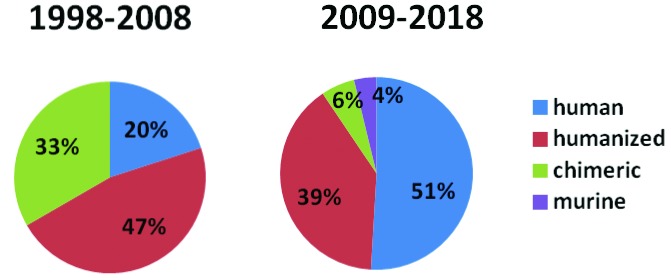
Distribution of monoclonal antibodies approved in the European Union by source.

**Figure 3:**
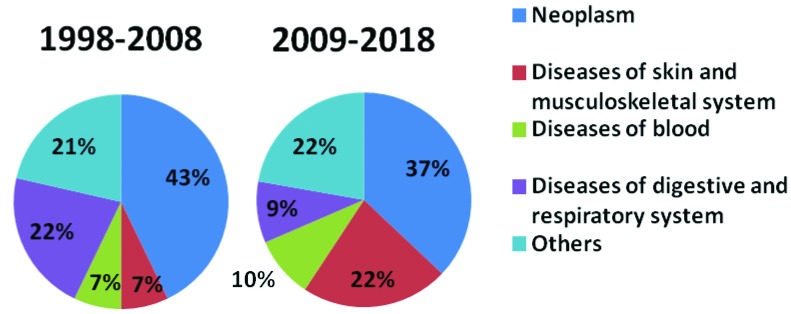
Distribution of monoclonal antibodies approved in the European Union by therapeutic target.

After a slow take-off, the number of mABs approved by the European Medicines Agency has dramatically increased in the last five years (Figure 1). The majority of mABs approved in the second decade are human or humanized ones (Figure 2), a trend which might reduce allergic reactions and boost clinical effects. The proportion of mABs indicated for cancer is still highest, although it has slightly decreased in the second decade (Figure 3).

The European public assessment reports for half of the 26 approved anti-cancer mABs do not list a biomarker (13) (Table 1). Biomarkers are reported more frequently for the mABs approved for solid tumors (14), which are slightly more numerous than those for blood cancers (12) – the latter being more curable than the former for a long time with small molecules. Of the nine biomarkers reported, all but one (Philadelphia chromosome) are proteins, and the only companion tests explicitly written in all the European reports are still those for detecting the biomarkers of the two “pioneer” targeted mABs (3).

**Table 1:** Anti-cancer monoclonal antibodies approved in the European Union, biomarkers indicated in their labels by European Medicines Agency, and main indications (1998-2018)*

International nonproprietary name†	Year of approval	Biomarker	Main indications
Rituximab	1998		non-Hodgkin lymphoma
Trastuzumab	2000	HER2^‡^	breast and stomach cancer
Cetuximab	2004	EGFR, KRAS and NRAS^‡^	colorectal cancer
Ibritumomab tiuxetan	2004		non-Hodgkin lymphoma
Bevacizumab	2005	EGFR^‡^	advanced non-small cell lung cancer
Panitumumab	2007	KRAS and NRAS^‡^	colorectal cancer
Ofatumumab	2010		chronic lymphocytic leukemia
Ipilimumab	2011		advanced melanoma and advanced renal cell carcinoma
Brentuximab vedotin	2012	CD30	Hodgkin lymphoma
Moxetumomab pasudotox	2013		B-lymphoblastic leukemia/lymphoma
Pertuzumab	2013	HER2^‡^	breast cancer
Obinutuzumab	2014		chronic lymphocytic leukemia
Ramucirumab	2014		gastric cancer
Blinatumomab	2015	Philadelphia-chromosome, CD19	acute lymphoblastic leukemia
Necitumumab	2015	EGFR	squamous non-small cell lung cancer
Nivolumab	2015		melanoma, non-small cell lung cancer
Pembrolizumab	2015	PD-L1	melanoma, non-small cell lung cancer, urothelial cancer, head and neck cancer
Daratumumab	2016		multiple myeloma
Elotuzumab	2016		multiple myeloma
Olaratumab	2016		soft tissue sarcoma
Atezolizumab	2017	PD-L1	urothelial cancer
Avelumab	2017		Merkel cell carcinoma
Inotuzumab ozogamicin	2017	CD22	acute lymphoblastic leukemia
Gemtuzumab ozogamicin	2018	CD33	acute myeloid leukemia
Mogamulizumab	2018		mycosis fungoides or Sézary syndrome
Durvalumab	2018	PD-L1	non-small cell lung cancer

*CD – cluster of differentiation; EGFR – epidermal growth factor receptor; HER2 – human epidermal growth factor receptor 2; PD-L1 – programmed death-ligand 1; KRAS – Kirsten RAt Sarcoma virus; NRAS – neuroblastoma RAS viral oncogene homolog.

^†^Source. Janice MR. The Antibody Society. Available from: https://www.antibodysociety.org/. Accessed: January 2019.

^‡^Companion test mentioned in the European Public Assessment Report.

Many of the more recent non-anti-cancer mABs have overlapping indications (Table 2). However, none of the 42 approved so far have a specific biomarker (and thus a companion test) indicated in the European reports.

**Table 2:** Non-anti-cancer monoclonal antibodies approved in the European Union and their main indications (1998-2018)

International nonproprietary name*	Approval year	Main indications
Abciximab	1995	prevention of cardiac ischemic complications, unstable angina
Basiliximab	1998	prevention of kidney transplant rejection
Palivizumab	1999	prevention of respiratory syncytial virus infection
Infliximab	1999	rheumatoid arthritis, Crohn's disease, psoriatic arthritis, psoriasis, ankylosing spondylitis
Adalimumab	2003	idiopathic arthritis, plaque psoriasis, Crohn's disease, uveitis
Omalizumab	2005	asthma
Natalizumab	2006	multiple sclerosis
Ranibizumab	2007	macular degeneration
Eculizumab	2007	paroxysmal nocturnal hemoglobinuria, atypical haemolytic uraemic syndrome
Certolizumab pegol	2009	rheumatoid arthritis, axial spondyloarthritis, psoriatic arthritis, plaque psoriasis
Ustekinumab	2009	Crohn's disease
Canakinumab	2009	periodic fever syndromes, Still's disease, Gouty arthritis
Golimumab	2009	rheumatoid and psoriatic arthritis, axial spondyloarthritis, ulcerative colitis
Tocilizumab	2009	rheumatoid arthritis
Denosumab	2010	osteoporosis
Belimumab	2011	systemic lupus erythematosus
Alemtuzumab	2013	multiple sclerosis
Raxibacumab	2014	anthrax infection
Siltuximab	2014	Castleman disease
Vedolizumab	2014	ulcerative colitis, Crohn's disease
Idarucizumab	2015	reversal of dabigatran-induced anticoagulation
Secukinumab	2015	plaque psoriasis
Mepolizumab	2015	severe eosinophilic asthma
Alirocumab	2015	hypercholesterolaemia, mixed dyslipidaemia
Evolocumab	2015	hypercholesterolaemia, mixed dyslipidaemia
Ixekizumab	2016	plaque psoriasis, psoriatic arthitis
Reslizumab	2016	asthma
Bezlotoxumab	2017	prevention of recurrent *Clostridium difficile* infection
Brodalumab	2017	plaque psoriasis
Guselkumab	2017	plaque psoriasis
Dupilumab	2017	atopic dermatitis
Sarilumab	2017	rheumatoid arthritis
Obiltoxaximab	2018	prevention of inhalational anthrax
Ocrelizumab	2018	multiple sclerosis
Emicizumab	2018	hemophilia A
Benralizumab	2018	asthma
Burosumab	2018	X-linked hypophosphatemia
Erenumab	2018	migraine prevention
Galcanezumab	2018	migraine prevention
Lanadelumab	2018	hereditary angioedema attacks
Tildrakizumab	2018	plaque psoriasis
Caplacizumab	2018	acquired thrombotic thrombocytopenic purpura

*Source: Janice MR. The Antibody Society. Available from: https://www.antibodysociety.org/. Accessed: January 2019.

## From theory to practice

### Precision medicine

Focused on biology rather than on other variables, such as lifestyle or environment, PM presents itself as the ultimate science, and its full promise goes even beyond targeting therapies for patients (14) and includes the ability to identify healthy individuals at high risk and take preventive measures for them (15). The PM’s basic assumption is that genetics is the underlying factor in most health conditions, so diseases are mainly affected by the human genetic make-up (16). Progress in characterizing individual differences in genomic sequences should extend the application range of PM from rare monogenic diseases to more common and genetically complex pathologies (17). Although diseases such as cancer and diabetes are largely a consequence of lifestyles, inherited genetic variations are considered their crucial cause (2).

Politically exploited by the former president of the USA to successfully baptize a major research initiative (2), PM has aroused great expectations as a “weapon” that will defeat most human diseases in the next decades and fully transform medicine from art to science (16,18). However, clinical results so far have not been as encouraging as promised. There is still a chasm between identifying a genetic susceptibility and developing safe and effective medicines. If no therapies are available, the value of diagnosis or risk stratification is very limited (5). Even more, combining various risk markers not necessarily implies providing clinically meaningful information, and much variability in therapeutic efficacy is not genetically driven (16). So, regardless of PM progress, complete success is still unlikely.

Especially for cancer, the major field of PM application, genetic mutations are numerous and evolve so heterogeneously in the majority of patients that it is almost impossible to find two identical tumors (19). This is why surgical interventions are still vital as they are the only way to immediately remove this cellular diversity from patients. There are persuasive scientific reasons why cancer has no miracle cure (20). When considered objectively, the potential of PM in oncology is sobering (21), with at best short-lived responses (and unavoidable toxicity) in a small proportion of patients, at high cost. Many of the recent successes against cancer still stem from traditional public health measures (eg, screening and early detection) (22). Although targeted therapies have so far offered limited benefit for overall survival − probably due to the adaptive nature of cancer (19,22) − PM still promises to pair patients with drugs based on genetic testing irrespective of the tissue of tumor origin (21).

### Monoclonal antibodies

Our survey on the mABs approved in the EU confirmed that in practice target prioritization is still a major issue, since in most cases there is a lack of biomarkers and the biomarkers are always the same in the few mABs that report them. After two decades, the industry’s mantra “no biomarker no drug” (11) has been mainly (for anti-cancer mABs) and fully (for the remaining ones) neglected in practice. Targeted therapies are still the major PM bottleneck, and “one size fits all” medicines continue to be employed (21). In oncology there are currently 343 mABs (60% of the total) under clinical trial (9), and the tendency is to treat tumors with different sites of origin with combination therapies (11,23) rather than to stratify therapies for the same cancer. This tendency should help further raise the already high returns generated by anticancer mABs through their sky-high prices (24), probably distorting further investment in this field at the expense of promising research in other disease areas.

## Comment

Critics argue that PM is continuously fueling unrealistic expectations, distracting funds from tackling widespread risk factors such as smoking, alcoholism, and obesity (5). Although it is well known that many diseases stem from unhealthy lifestyles and socio-economic conditions (22), researchers’ ambitions and media channels relentlessly foster the arguable PM promises, and keep attracting big funding (14). However, PM advocates would do better to temper their narrative of radical change and communicate a more realistic set of expectations through the media to the public (5), in line with the incremental nature of science.

While we are waiting for the gap between the exorbitant expectations raised by PM and the discouraging results achieved so far to be filled, there is widespread evidence in this period of never-ending economic crisis that pharmaceutical expenditure has become increasingly unsustainable for health authorities even in most high-income countries (25), and Europe is no exception.

Pharmaceutical expenditure, like anything else, is determined by prices and volumes, and the former are increasingly out of control. This was easy to predict in a typically “market failure” situation (26), where prices cannot competitively match demand with supply. Since all pharmaceutical prices are necessarily set through arbitrary decisions, the unavoidable results are a distortion of relative prices and irrational allocation of financial resources in pharmaceuticals (27), from upstream research investments to downstream health expenditures.

The pharmaceutical industry is mainly private, and negotiating sky-high prices for new drugs like mABs is a crucial factor of success when it comes to generating high returns on research and development investments and maximizing profits in all countries (28). In this landscape, PM helps create an ideal setting for price discrimination for new similar drugs (29), and anti-cancer mABs can be considered an emblematic example of less and less sustainable prices (30). Because of the emotive nature of cancer, health authorities find it hard to resist “pleas” for reimbursement of new drugs, even when their efficacy is marginal (31). So, pharmaceutical companies have a clear incentive to invest in new anti-cancer therapies, regardless of their real impact on patients’ survival and quality of life (32).

We contend here that the time has come to stop setting arbitrary prices for new, very expensive drugs, so as to limit the distortion of allocation of financial resources in pharmaceuticals (33). Prices can hardly − if ever − be competitive in a “market failure” context, so their effect should be minimized. Leveling out prices for a very limited number of therapeutic classes and capping expenses to respect yearly budgets should become the “recipe” to master pharmaceutical expenses in the future (33). It is hard enough – probably impossible − to rank therapies on the basis of the importance of their related pathologies, so the benefits of effective medicines are even harder to differentiate through pricing.

If we strive to restore a balance between public objectives of health services and the private incentives of the pharmaceutical industry, and improve the long-term sustainability of pharmaceutical expenditure in all European countries, rational budgeting should be given priority over irrational pricing. Realistic expectations to improve patients’ health thanks to PM as a concept and mABs as therapies might then be pursued in the long run with much less suspicion in the literature.

## References

[1] Psaty BM, Dekkers OM, Cooper RS (2018). Comparison of 2 treatment models: precision medicine and preventive medicine.. JAMA.

[2] Collins FS, Varmus H (2015). A new initiative on precision medicine.. N Engl J Med.

[3] Garattini L, Curto A, Freemantle N (2015). Personalized medicine and economic evaluation in oncology: all theory and no practice?. Expert Rev Pharmacoecon Outcomes Res.

[4] Tutton R (2012). Personalizing medicine: futures present and past.. Soc Sci Med.

[5] Evans JP, Meslin EM, Marteau TM, Caulfield T (2011). Genomics. Deflating the genomic bubble.. Science.

[6] Conti R, Veenstra DL, Armstrong K, Lesko LJ, Grosse SD (2010). Personalized medicine and genomics: challenges and opportunities in assessing effectiveness, cost-effectiveness, and future research priorities.. Med Decis Making.

[7] Vegter MW (2018). Towards precision medicine; a new biomedical cosmology.. Med Health Care Philos.

[8] Yan L, Beckman RA (2005). Pharmacogenetics and pharmacogenomics in oncology therapeutic antibody development.. Biotechniques.

[9] Kaplon H, Reichert JM (2019). Antibodies to watch in 2019.. MAbs.

[10] Godman B, Finlayson AE, Cheema PK, Zebedin-Brandl E, Gutiérrez-Ibarluzea I, Jones J (2013). Personalizing health care: feasibility and future implications.. BMC Med.

[11] de Bono JS, Ashworth A (2010). Translating cancer research into targeted therapeutics.. Nature.

[12] Garattini L, van de Vooren K, Curto A (2015). Cost-effectiveness of trastuzumab in metastatic breast cancer: mainly a matter of price in the EU?. Health Policy.

[13] European Medicines Agency. European public assessment reports available from: https://www.ema.europa.eu/en/medicines/field_ema_web_categories%253Aname_field/Human/ema_group_types/ema_medicine. Accessed: May 10, 2019.

[14] Ten Have H, Gordijn B (2018). Precision in health care.. Med Health Care Philos.

[15] Dzau VJ, Ginsburg GS, Van Nuys K, Agus D, Goldman D (2015). Aligning incentives to fulfil the promise of personalised medicine.. Lancet.

[16] Wiesing U (2018). From art to science: a new epistemological status for medicine? On expectations regarding personalized medicine.. Med Health Care Philos.

[17] Ehmann F, Caneva L, Prasad K, Paulmichl M, Maliepaard M, Llerena A (2015). Pharmacogenomic information in drug labels: European Medicines Agency perspective.. Pharmacogenomics J.

[18] Tooke J (2016). The science (and art) of medicine.. Lancet.

[19] Swanton C (2018). Cancer therapeutics through an evolutionary lens.. J R Soc Med.

[20] Abbasi K (2018). The seductive hope of cancer therapies.. J R Soc Med.

[21] Prasad V (2016). Perspective: The precision-oncology illusion.. Nature.

[22] Joyner MJ, Paneth N (2015). Seven questions for personalized medicine.. JAMA.

[23] Wise J (2019). Genome sequencing of children promises a new era in oncology.. BMJ.

[24] Tay-Teo K, Ilbawi A, Hill SR (2019). Comparison of sales income and research and development costs for fda-approved cancer drugs sold by originator drug companies.. JAMA Netw Open.

[25] Ploumen L, Schippers E (2017). Better life through medicine − let’s leave no one behind.. Lancet.

[26] Garattini L, Padula A (2018). Competition in pharmaceuticals: more product- than price-oriented?. Eur J Health Econ.

[27] Frakt AB, Chernew ME (2018). The Importance of relative prices in health care spending.. JAMA.

[28] Garattini L, Padula A. Between pharmaceutical patents and European patients: is a compromise still possible? Expert Opin Ther Pat. 2017; 27(10):1073-6. 10.1080/13543776.2017.1350648 28671001

[29] Chandra A, Garthwaite C (2017). The economics of indication-based drug pricing.. N Engl J Med.

[30] Dolgin E (2018). Bringing down the cost of cancer treatment.. Nature.

[31] van de Vooren K, Curto A, Garattini L (2013). Optional copayments on anti-cancer drugs.. BMJ.

[32] Reinhardt U (2015). Probing our moral values in health care: the pricing of specialty drugs.. JAMA.

[33] Garattini L, Padula A (2018). Pharmaceutical pricing conundrum: time to get rid of it?. Eur J Health Econ.

